# Discovery of novel inhibitors of *Streptococcus pneumoniae *based on the virtual screening with the homology-modeled structure of histidine kinase (VicK)

**DOI:** 10.1186/1471-2180-9-129

**Published:** 2009-06-27

**Authors:** Nan Li, Fei Wang, Siqiang Niu, Ju Cao, Kaifeng Wu, Youqiang Li, Nanlin Yin, Xuemei Zhang, Weiliang Zhu, Yibing Yin

**Affiliations:** 1 Key Laboratory of Laboratory Medical Diagnostics, Ministry of Education, Faculty of Laboratory Medicine, Chongqing Medical University, Chongqing 400016, PR China; 2 Drug Discovery and Design Centre, State Key Laboratory of Drug Research, Shanghai Institute of Material Medical, Graduate School of Chinese Academy of Sciences, Shanghai 201203, PR China

## Abstract

**Background:**

Due to the widespread abusage of antibiotics, antibiotic-resistance in *Streptococcus pneumoniae *(*S. pneumoniae*) has been increasing quickly in recent years, and it is obviously urgent to develop new types of antibiotics. Two-component systems (TCSs) are the major signal transduction pathways in bacteria and have emerged as potential targets for antibacterial drugs. Among the 13 pairs of TCSs proteins presenting in *S. pneumoniae*, VicR/K is the unique one essential for bacterium growth, and block agents to which, if can be found, may be developed as effective antibiotics against *S. pneumoniae *infection.

**Results:**

Using a structure-based virtual screening (SBVS) method, 105 compounds were computationally identified as potential inhibitors of the histidine kinase (HK) VicK protein from the compound library SPECS. Six of them were then validated *in vitro *to be active in inhibiting the growth of *S. pneumoniae *without obvious cytotoxicity to Vero cell. In mouse sepsis models, these compounds are still able to decrease the mortality of the mice infected by *S. pneumoniae *and one compound even has significant therapeutic effect.

**Conclusion:**

To our knowledge, these compounds are the first reported inhibitors of HK with antibacterial activity *in vitro *and *in vivo*, and are novel lead structures for developing new drugs to combat pneumococcal infection.

## Background

*S. pneumoniae *is a major risk factor with high morbidity and mortality world-widely, especially in the elderly and children. It is believed to be one of the four major infectious disease killers [1-5]. Meanwhile, an increasing number of bacterial strains with resistance are encountered in the clinic nowadays, among which antibiotic-resistant *S. pneumoniae *has caused many deaths due to antibiotics abusage in hospitals. Therefore, it is urgent to develop new types of antibiotics.

In prokaryotes, the two-component signaling systems (TCSs), each pair of which are typically composed of histidine kinase (HK) and response regulator (RR), play important roles in drug-resistance, pathogenesis and bacterial growth [6-8]. The regulation of TCS on histidine phosphorylation in signal transduction distinct from that on serine/threonine and tyrosine phosphorylation in higher eukaryotes [9]. For some TCSs, both the HK and RR are essential for bacterial viability in several Gram-positive pathogens, including *Bacillus subtilis *(*B. subtilis*), *Enterococcus faecalis *and *Staphylococcus aureus *(*S. aureus*) [10-13], and thus received attention as potential targets for antimicrobials [9,14-17]. In *S. pneumoniae*, although at least 13 TCSs were identified, only TCS02 (also designated as VicR/K [18], MicA/B [19] or 492 hk/rr [20]) is essential for bacteria viability, which can be a potential target for antimicrobial intervention. To be detailed, in TCS02, only functional VicR appears to be essential for *S. pneumoniae *[21], without which *S. pneumoniae *can't grow or act as a pathogen [22]. However, the crystal structure of VicR is unsuitable for structure-based virtual screening because the active site is too shallow to dock a small molecule [22,23]. The reason that VicK does not seem to be essential for *S. pneumoniae *viability, was supposed to be that some currently unknown HKs also participate in the activation of VicR by phosphorylation [24,25]. However, among these HKs, VicK it is best-known one with definite action on VicR. Moreover, recent researches showed a high-degree homology in the catalytic domain of these HKs [14-17]. Thus theoretically, selective inhibitors to VicK, a representative of HKs, can interrupt the phosphorylation of VicR and ultimately reduce the viability of *S. pneumoniae*.

The structure-based virtual screening (SBVS), an approach used widely in drug design and discovery, possesses many advantages, such as rapidness, economization, efficiency and high-throughput. In the recent years, SBVS has attracted great attention in developing innovative antimicrobial agents. A case in point is the discovery of a lead-compound named diarylquinoline against *Mycobacterium tuberculosis *[26]. Our study here was designed to search the compound database for potential inhibitors targeting the VicK protein of *S. pneumoniae *by using *in silico *and experimentalmethods, which may provide much valuable information to develop new antibiotics against pneumococcal infection.

## Results

### Sequence analysis of the VicK TCS in *S. pneumoniae*

Domain analysis http://smart.embl.de/smart/show_motifs.pl?ID=Q9S1J9 indicated that the VicK protein of *S. pneumoniae *contained one transmembrane segment and several domains: PAS, PAC, HisKA and HATPase_c. Multi-alignment of the HATPase_c domain sequences showed that in most bacteria the sequences around the ATP binding site of VicK HKs are similar and have four conserved motifs: the N box, G1 box, F box and G2 box [27]. This high homology of ATP binding domain of HKs in bacteria makes it reasonable to screen antibacterial agents by using this domain as a potential target [16].

**Figure 1:**
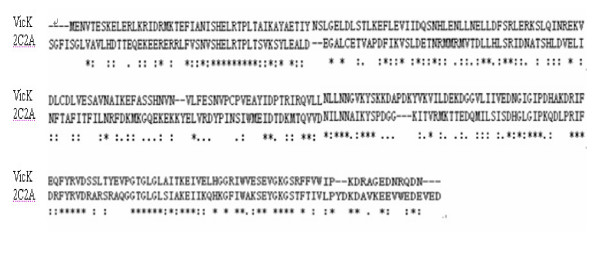
**The sequence alignment of the HATPase_c domain of VicK in *S. pneumoniae *and **2c2a. The symbols below the alignment represent the similarity between two proteins. "*" denotes identical residues between two sequences, ":"means similar residues, "." means a bit different and blank means completely different. Schematic alignment diagram was made by the program ClustalX.

Compared with VicK HATPase_c domain in *S. pneumoniae *(GenBank accession number: AAK75332.1), the most homologous sequence in the structural Protein Data Bank (PDB) was the similar domain of *Thermotoga maritime *(PDB entry: 2c2a) [28], a TCS molecule, with 33% sequence identity and 57% conservative replacements (Figure 1). This domain is the entire cytoplasmic portion of a sensor HK protein. The X-ray crystal structure of the domain of *Thermotoga maritima *was therefore used as a template for modeling the 3D structure of the VicK HATPase_c domain of *S. pneumoniae*.

### A 3D model of the VicK HATPase_c domain of *S. pneumoniae*

**Figure 2:**
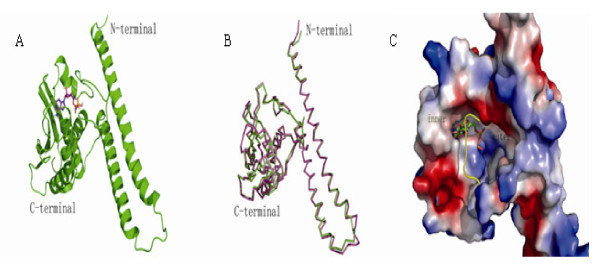
**The modeled structure of the VicK HATPase_c domain of *S. pneumoniae***. (A) The solid ribbon representation of the structure model of the VicK HATPase_c domain. (B) Structure superposition of sketch of modeled VicK structure with the template. (C) Shape and surface features of the ATP-binding pocket of the VicK HATPase_c domain. The color denotes electrostatic potential of the protein surface. The red and blue color show negative and positive charged potential respectively, and the white surface means neutral potential of non-polar hydrophobic residues. The ATP-binding pocket is divided into "inner" and "outer" parts. The loop covered on the pocket is shown as tube for the sake of clearly demonstrating the hydrophobic inner part. The outer part of pocket is hydrophilic because of many polar residues in the entrance of the pocket, including the polar loop structure. All the pictures were generated by PyMol http://www.pymol.org/.

Based on the X-ray diffraction crystal structure of the homologous domain of the *Thermotoga maritima*, a 3D model for the VicK HATPase_c domain of *S. pneumoniae *was constructed. Figure 2A shows the final structure of this model that were checked and validated using structure analysis programs Prosa and Profile-3D [29]. This model of 3D structure contains five stranded β-sheets and four α-helices, which form a two-layered α/β sandwich structure. Figure 2B indicates that the model superposed well with the homologous domain of *Thermotoga maritima*, with a root-mean-square deviation (RMSD) of the Cα atoms being about 1.34 Ǻ. The surface shape and general electrostatic feature of the HATPase_c domain of VicK were shown in Figure 2C. The ATP binding site consists of a relatively hydrophobic inner cavity and a larger hydrophilic outer cavity. Both cavities are connected by a gorge-like channel, and are consisted of highly conserved residues which can bind and fix the substrate. The inner part lack of polar amino acid residues can accommodate the adenosine, while the outer one rich in charged residues can bind the triphosphate.

### Discovery of potential inhibitors of the *S. pneumoniae *VicK HK by virtual screening

The target site for high throughput virtual screening (HTVS) was the ATP-binding pocket of the VicK HATPase_c model of *S. pneumoniae*, which consisted of residues within a radius of 4 Ǻ around the ATP site. In the primary screening, the database SPECS containing about 200,000 molecules was searched for potential binders using the program DOCK4.0 [30,31]. Subsequently, structures ranked in the first 10,000 were re-scored by using the Autodock 3.05 program [32]. As a result, about 200 molecules were filtered out by these highly selective methods. Finally, we manually selected 105 molecules according to their molecular diversity, shape complementarities, and the potential to form hydrogen bonds and hydrophobic interactions in the binding pocket of the VicK HATPase_c domain.

### Inhibition of the VicK' protein ATPase activity in vitro

**Figure 3:**
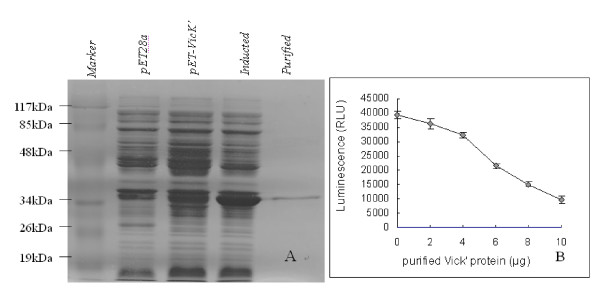
**(A) SDS-PAGE analysis of VicK' purification (B) Identification of kinase activity of VicK' protein *in vitro***. Variant amounts of VicK' proteins were added into reaction systems containing a constant ATP concentration (5 μM). Each assay was performed in quadruplicate and repeated three times. Luminescent output is inversely correlated with the concentration of the kinase.

In order to confirm the interaction of the potential VicK inhibitors with their putative target protein, we expressed and purified His-tagged VicK' protein by using the pET28a plasmid in BL21(DE3) as shown in Figure 3A. The kinase activity of VicK' protein was measured by quantifying the amount ATP remained in solution after the enzymatic reaction (Figure 3B). These results indicated that the purified VicK' protein possessed the ATPase activity, which can hydrolyze ATP *in vitro*. Using the purified active VicK', we obtained 23 compounds from the 105 candidate inhibitors which could decrease the ATPase activity of VicK' protein by more than 50%, indicating these compounds may also be potential VicK inhibitors in *S. pneumoniae*.

### Antimicrobial activities of potential VicK' inhibitor and Cytotoxicity of the antimicrobial compounds in vitro

**Figure 4:**
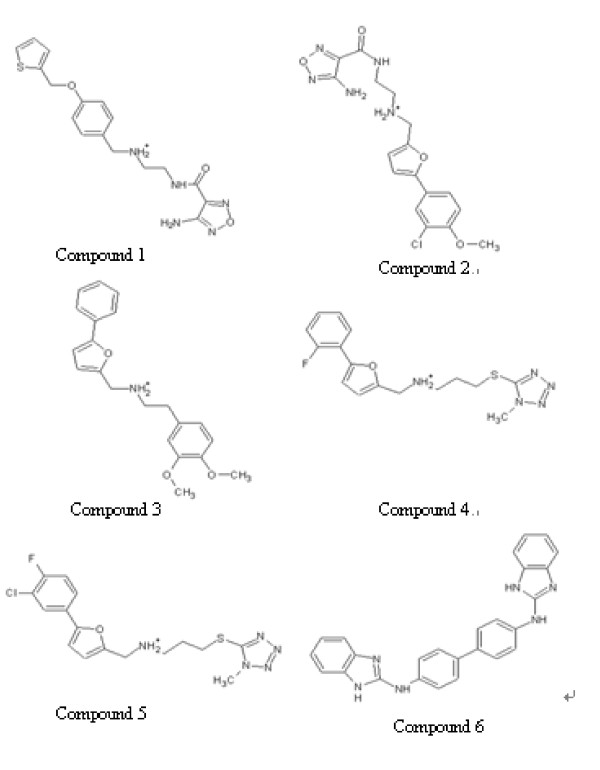
**Chemical structures of the compounds with inhibitive effects on the growth of *S. pneumoniae***. These six inhibitors belong to three different classes of chemical structures: one imidazole analogue (compound 6), four furan derivatives (compound 2, 3, 4 and 5) and one derivative of thiophene (compound 1).

**Figure 5:**
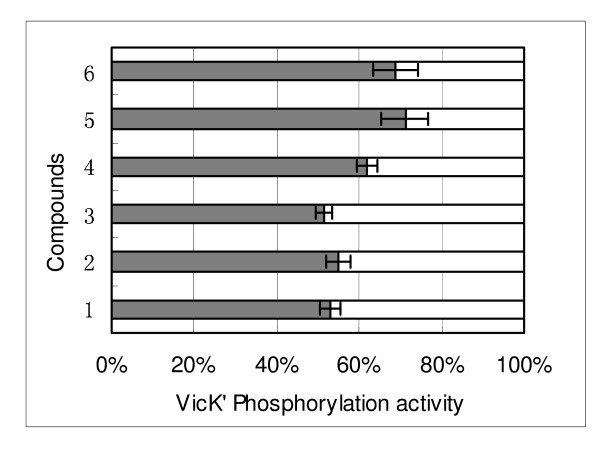
**Inhibition ratio of VicK' protein autophosphorylation by six lead compounds with antibacterial effects (from the 23 compounds)**. The inhibitory activities of the compounds for the ATPase activity of the VicK' protein was measured using the Kinase-Glo™ Luminescent Kinase Assay. Briefly, purified VicK' protein(6 μg/50 μl) was pre-incubated with compounds(final concentration, 200 μM) in a reaction buffer containing 40 mM Tris-HCl (pH 7.5), 20 mM MgCl_2 _and 0.1 mg/ml BSA, at room temperature for 10 min. Then ATP (5 μM) was added for another incubation of 10 min at room temperature, and detected the rest amount of ATP.

We investigated the bactericidal activity of these 23 compounds against *S. pneumoniae *using a standard minimal bactericidal concentration assay (MIC) (Table 1). Six compounds (Figure 4), each inhibiting the VicK' activity by more than 50% (52.8%, 54.8%, 51.6%, 61.9%, 71.1% and 68.8%, respectively) (Figure 5), could obviously inhibit the growth of *S. pneumoniae*, with MIC values below 200 μM. Moreover, their MIC values were positively correlated with the corresponding IC_50 _(the concentration of inhibiting 50% VicK' protein autophosphorylation) values (r = 0.93), which indicates that the bactericidal effects of these chemicals were realized by disrupting the VicK/R TCS system in *S. pneumoniae*. Chemical structures of these 6 compounds are shown in Figure 4, which belong to three different classes of chemicals: one imidazole analogue, four furan derivatives and one derivative of thiophene (Figure 4).

**Table 1:** Biological effects of six potential inhibitors of the VicK histidine kinase

Chemical inhibitor	MIC (μM)	MBC (μM)	CC_50 _(μM) on Vero cell	IC_50 _(μM) for VicK' protein
Compound 1	**100**	**>200**	213	542.25
Compound 2	**50**	**200**	321.33	562.41
Compound 3	**100**	**>200**	274.22	502.63
Compound 4	**200**	**>200**	360	>1000
Compound 5	**100**	**>200**	516.17	598.11
Compound 6	**0.28**	**25**	392	32.60
PNC	**0.02**	**2.0**	undone	undone

A 3-(4, 5-dimethylthiazol-2-yl)-2, 5-diphenyl tetrazolium bromide (MTT) assay was carried out on Vero cell line to determine the CC_50_(concentration that induces a 50% cytotoxicity effect) values of these compounds. As shown in Table 1, the CC_50 _values of all these six compounds were larger than 200 μM and than their respective MIC values, indicating low cytotoxicity effects on Vero cell. Collectively, these compounds inhibited bacterial growth with low toxic effects.

### Time- and concentration-dependent growth curve

**Figure 6:**
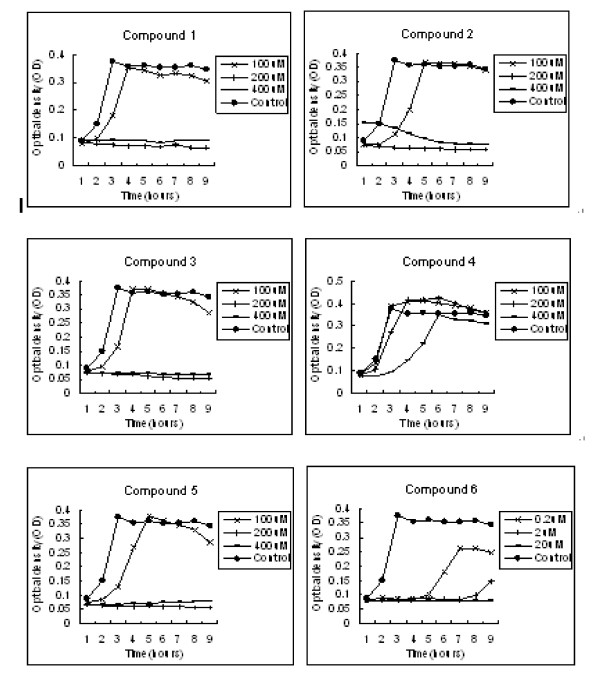
**Time and concentration-dependent effects of the candidate compounds on the growth of *S. pneumoniae in vitro***.

While several compounds identified in our study could be used as excellent drug leads *in vitro*, the best and most valuable ways would be *in vivo *validation. The following results of the time- and concentration-dependent effects of the lead inhibitors on the growth of *S. pneumoniae *further illustrated their antibacterial characteristics, and would be an important guide for *in vivo *administration. As shown in Figure 6, the similar curves of compounds 1, 2, 3 and 5 indicated that these compounds have significant activity against *S. pneumoniae *at concentration of about 200 μM, and this activity could last at least 8 hours. The most efficient inhibitor identified was compound 6, which had bactericidal effect against *S. pneumoniae *even at concentration of as low as 0.2 μM. However, even at concentration of 400 μM, compound 4 was not likely to have bactericidal effect, but it seemed to have delayed the multiplication of *S. pneumoniae*.

### Therapeutic effects of the lead compounds in mouse S. pneumoniae infections

**Figure 7:**
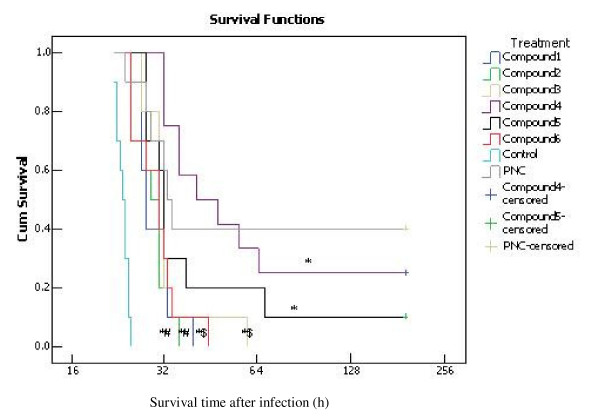
**Therapeutic efficacies of each lead compound against infection with *S. pneumoniae *ATCC7466 in mice**. Figure shows the cumulative survival (survival probability) of the mouse infection models treated differently in the following 8 days (survival time more than 8 days was censored). Data were analyzed by using the survival analysis approach (Kaplan-Meier Method). Significant treatment effects were found among the groups (P < 0.01) by an overall comparison. Pairwise comparisons revealed that compounds 1–6 prolonged survival time in mouse infection models as compared to negative control (*p *< 0.01), and that compound 4 and 5 were almost as effective as positive control PNC (P > 0.1), but the other compounds were less effective than it (P < 0.05 or P < 0.1). *P < 0.01 indicates significant differences as compared to negative control; ^#^P < 0.05 and ^$^P < 0.1 indicate significant differences as compared to positive control.

Mouse sepsis models by *S. pneumoniae *(ATCC 7466) were successfully established by intraperitoneal injection of 100 μl *S. pneumoniae *(5 × 10^3 ^CFU/ml). Generally, these mice began to die within 24 hours and couldn't survive more than 48 hours unless they got appropriate therapeutic treatments. For facilitation of comparisons between the effects of these compounds and positive control (penicillin), the concentration of penicillin used in this study almost equaled to that of the lead compounds. To rule out the direct antibacterial effects that may compromise with the efficiency of this model, the lead compounds and penicillin were administrated through caudal vein. As shown in Figure 7, these compounds were able to decrease, though slightly, the mortality of the infected mice in the first 24 hours as compared to negative control (normal sodium, NS) (*p *< 0.01). Significant treatment effects were found among the groups (*p *< 0.01) by an overall comparison. Pairwise comparisons revealed that compounds 1–6 prolonged survival time in mouse sepsis models as compared to negative control (*p *< 0.01). However, compound 1, 2, 3 and 6 were less effective than positive control PNC (*p *< 0.05 or *p *< 0.1). Although these compounds could not reverse the fatal pneumococcal infection with concentration used in this study, *in vivo *antibacterial activity of these six compounds suggested that it would be promising to develop lead-compound-based drugs against pneumococcal infection.

### Molecular modeling of VicK' protein and its potential inhibitors

**Figure 8:**
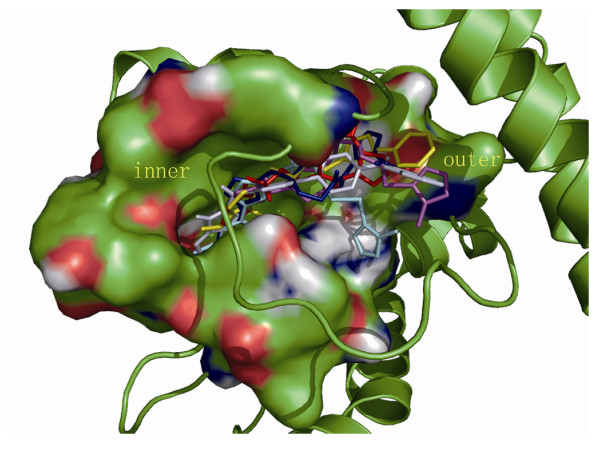
**Three-dimensional structural binding modes of six potential inhibitors to VicK' protein derived from the docking simulations**. The loop covered on the pocket was shown in tube. Six compounds were shown in stick with different colors. Their binding conformations showed similar interaction modes in the inner pocket. The binding diversity was restrained by small space and hydrophobic characteristic. By contrast, these structures bound in the outer pocket in various ways. This image was generated using the PyMol program http://www.pymol.org/.

In order to get insight into the mechanism of inhibition, further studies were carried out to verify the interaction modes between six compounds and the modeled structure of VicK' protein. Autodock 3.05 software was used for the docking simulation. The binding conformations of these inhibitors in the ATP-binding pocket of the VicK HATPase_c domain were shown in Figure 8. Although these structures are diverse, the binding models of six potential inhibitors are similar, especially in the inner part of the conserved domain. The surface of the binding pocket (Figure 2C) is divided into two parts, one is hydrophobic inner part composed of residues ILE146, ILE175, LEU180, ILE182, PHE238, and the other is the outer hydrophilic part consisted of residues ASN149, LYS152, TYR153, ARG196, ARG199. All six compounds bind in the pocket with rigid aromatic ring parts inserting into the inner part. In the large and flexible outer part, these compounds adopt different interactions. All of them have hydrogen bond acceptors in the binding outer part. They could form hydrogen networks with the polar residues to stabilize the substrate interactions. Their binding models resemble natural substrate ATP much.

## Discussion

In bacteria, HKs have fundamental roles in TCS signal transduction pathways. Thus they are major targets for antibacterial drug development. High structural and sequence homology of this kinase gene family makes the HKs ideal targets for homology modeling and structure based virtual screening. SBVS is an approach based on the three-dimensional structures of macromolecular to identify chemical entities binding to the targets and to elicit potential biological mechanisms with the advantages of speed, efficiency and high-throughput. The availability of the small molecular lead-compound library and the modeled 3D target structure makes it possible to use SBVS to screen out a limited number of promising candidates that can interrupt the TCS signal transduction by interacting with the HKs substrate of *S. pneumoniae*.

HKs, as novel antibacterial targets, have attracted many attentions due to their essentiality in the viability of microbes and their deficiency in animals. HKs are involved in the regulation of bacterial growth and virulence in many bacterial species. Previously, a HK named VicK has been used to screen lead compound inhibitors in *B. subtilis *and *S. epidermidis*. We here for the first time obtained 105 candidate chemical compounds directly aiming at *S. pneumoniae *VicK by screening 200,000 possible compounds *in silico*. Compounds that can bind to the purified target protein VicK' and compete with its substrate ATP were further verified by *in vitro *and *in vivo *antibacterial assays. Eventually, we obtained 6 compounds with antibacterial activity that may be used as novel drug leads.

Commonly, the response regulator YycF and the histidine kinase YycG are the only essential TCS for viability in *B. subtilis *and *S. aureus *[10,12]. In *S. pneumoniae*, the VicR/K TCS regulates the expression of several critical genes, such as those encoding surface proteins and virulence factors [21,33]. However, only the response regulator VicR was found to be essential [20,34]. The signal transduction of VicK was possibly bypassed by other TCS HKs [35]. VicK has conserved ATP-dependent HATPase_c domains accounting for autophosphorylation. Even non-cognate HKs from other bacteria can phosphorylate the purified VicR from *S. pneumoniae *[18]. In a previous study [36], the MIC values of the lead compounds screened out by SBVS targeting the YycG of *S. epidermidis *were almost equal to the corresponding IC_50 _(for YycG') values, with a correlation coefficient of 0.959, which suggested that inhibition of 50% the YycG protein activity would interfere with the growth of *S. epidermidis*. If this case is true in *S. pneumoniae*, the result that the MIC values of the lead-compounds were far less than the corresponding IC_50 _values may be explained as bypass effects of these compounds on other HKs. In a word, these lead compounds are most likely having a "cross-inhibition" on other HKs in *S. pneumoniae*, which can enhance their antibacterial effects, although they were not verified in this study.

Although the VicK protein in *S. pneumoniae *can be homologous to YycG in other Gram-positive strains, such as *S. epidermidis*, *Enterococcus faecalis *and *S. aureus*, different strains generally have different characteristics of the HATPase_c domain structure of HKs. These characteristics will determine the binding specificity of the lead compounds screened out by SBVS. Moreover, a different template for homologous modeling and different parameters for SBVS were used, which can guarantee the specificity of the lead compounds binding to the VicK' discovered. What's more, 23 compounds can inhibit the purified VicK' protein activity by more than 50%, 6 of which displayed different degrees of antibacterial effects *in vitro *and *in vivo*. Regretfully, the *in vivo *activities of these compounds were not quite consistent with their corresponding *in vitro *activity, and some compounds displayed obvious cytotoxicity, which would challenge our future investigation. Moreover, it seems to be a paradox that compound 4 have less bactericidal effects in the time- and concentration-dependent antibacterial assays, but demonstrated significant therapeutic effects in mice infected by *S. pneumoniae*. However, due to the VicK' is not essential in *S. pneumoniae*, this chemical may have a possibility to interrupt the invasion and virulence rather than cause numerous death of the bacterium, which decreases the selection pressure and contributes to the maintenance of species diversity, thus reduces the emergence of drug-resistant strains. Anyway, the subtle mechanisms need our future work.

## Conclusion

To summarize, we have successfully found out several promising lead compounds for further drug development in this study, which also can be used as inhibitors to explore the mechanism of autophosphorylation by VicK as well as other HKs. Important work in future would be validation of their antibacterial effects in different strains and structural modification for more effective derivatives with less *in vivo *toxicity, and investigation into whether they can bind to other ATP-dependent kinase is also necessary.

## Methods

### Bacterial strains, media and reagents

*S. pneumoniae *(D39) ATCC 7466 was purchased from the American Type Culture collection (ATCC, USA).*S. pneumoniae *D39 was grown in C + Y medium. Plasmids were transformed into *Escherichia coli *(*E. coli*) strains that were grown in Luria-Bertani (LB) broth. For selection of *E. coli *transformants, kanamycin (50 μg/ml, final concentration) was added to the growth medium.

All compounds screened out in our study were purchased from the SPECS Company in the Netherlands. Stock solutions of the compounds were prepared in Dimethyl Sulfoxide (DMSO). Other chemicals were purchased from Sigma.

### Bioinformatics analysis

Domain analysis was performed based on the SMART database. The complete genome sequences of the *S. pneumoniae *strain ATCC 7466 were accessed from the National center for Biotechnology information (NCBI) genome database. For the homologous sequences with the VicK HATPase_c domain of *S. pneumoniae *ATCC 7466, the Protein Data Bank (PDB) was searched by using the Blastp program. ClustalX was used to align the protein sequences.

### 3D structure modeling of the VicK HATPase_c domain

The sequence of *S. pneumoniae *VicK was retrieved from GenBank (accession number: AAK75332.1). The Align123 module in Insight II was used in the pairwise sequence alignment. Using the secondary structure information of *Thermotoga maritima *(PDB entry: 2c2a), the sequence alignment was adjusted manually to obtain a fine alignment for 3D structure construction. The 3D model of the VicK HATPase_c domain was generated by using the MODELLER module in Insight II. Several structural analysis programs such as Prostat and Profile-3D were used to check the structure quality. The Prostat module of Insight II was used to analyze the properties of bonds, angles, and torsions. The profile-3D program was used to check the structure and sequence compatibility.

### Structure-based virtual screening

Structure-based virtual screening was performed as described previously [36], with modification. Briefly, the binding pocket of the VicK HATPase_c domain was used as a target for screening the SPECS database by using the docking approach. A primary screening was conducted by using the program DOCK4.0. Residues within a radius of 4 Ǻ around the ATP-binding pocket of the VicK HATPase_c domain were used for constructing the grids for the docking screening. Subsequently, the 10,000 compounds with the highest score as obtained by DOCK search were selected for a second round docking by using the Autodock 3.05 program, followed by our own filter of druglikeness to eliminate the non-drug-able molecules. Finally, we manually selected 105 molecules according to their molecular diversity, shape complementarities, and potential to form hydrogen bonds in the binding pocket of the VicK HATPase_c domain.

### Molecular modeling of the interaction between inhibitors and the target protein

To determine the binding modes, Autodock3.05 was used for automated docking analysis. The Lamarchian genetic algorithm (LGA) was applied to deal with the protein-inhibitor interactions. Some important parameters were set as follows: the initial number of individuals in population is 50; the elitism value is 1, which automatically survives into nest generation. The mutation rate is 0.03, which is a probability that a gene would undergo a random change. The crossover rate, the probability of proportional selection, is 0.80. Every compound was set to have 10 separated GA runs and finally 10 conformations would be generated. The conformations were clustered automatically and the conformation with minimum binding free energy in the cluster with minimum RMSD value was selected as the representative conformation of the inhibitor.

### Cloning, expression and purification of the VicK protein

The VicK gene fragment containing the cytoplasmic signal domains (the HATPase_c and HisKA domain) of VicK (coding 200–449 aa) was amplified by PCR. The upstream and the downstream primers were 5'-CGGGATCCGAGCAGGAGAAGGAAGAAC-3' and 5'-CGCTCGAGGTCTTCTACTTCATCCTCCCA-3' respectively. Subsequently, the fragment was digested with *EcoR *I and *Xho *I (TaKaRA, Japan) and ligated into the corresponding sites of pET28a to obtain a recombinant plasmid pET28/VicK'. After being transformed into *E. coli *strain BL21 (DE3), this recombinant plasmid was induced to express the protein of VicK' by 0.2 mM isopropyl-1-thio-β-D-galactopyranoside (IPTG) at 24°C for 20 hours. Cells were harvested and sonicated, and then the debris was removed by centrifugation. The fraction containing the cytoplasmic domain was isolated from the supernatant solution through a His-tagged column, with a purity of more than 95%, as assessed by gel electrophoresis and Coomassie Blue staining.

### Inhibition assay for the ATPase activity

The inhibitory activity of the compounds for the ATPase activity of the VicK' protein was measured using the Kinase-Glo™ Luminescent Kinase Assay (Promega, Madison, USA). Briefly, 6 μg purified VicK' protein was pre-incubated with a series of dilutions of compounds in a reaction buffer containing 40 mM Tris-HCl (pH 7.5), 20 mM MgCl_2 _and 0.1 mg/ml BSA, at room temperature for 10 min. Then 5 μM ATP was added for another incubation of 10 min at room temperature, and Kinase-Glo™ Reagent was added to detect the rest amount of ATP, as reflected by luminescence intensity (Lu). In parallel, the VicK' protein with no addition of compounds was used as control and ATP only was used as blank. The rate of inhibiting protein phosphorylation (R_p_) by the compounds was calculated by the following equation: R_p _= (Lu_compound _- Lu_control_)/(Lu_blank _- Lu_control_) × 100%. IC_50 _(the concentration of inhibiting 50% VicK' protein autophosphorylation) was calculated by using the SPSS 11.0 software.

### Minimal inhibitory concentration (MIC) and minimal bactericidal concentration (MBC) assays

MIC assays for the antibacterial activities of the compounds were performed according to the broth micro-dilution (in 96-well plate) methods of the Clinical and Laboratory Standards Institute (CLSI) of America. The Minimal Bactericidal Concentration (MBC) was obtained by sub-culturing 200 μl from each negative (no visible bacterial growth) well in the MIC assay which were then plated onto Columbian blood plates. The plates were incubated at 37°C for 24 hours, and the MBC was defined as the lowest concentration of substance which produced subcultures growing no more than five colonies on each plate. Each assay was repeated at least three times.

### Time- and concentration-dependent curve

*S. pneumoniae *strains ATCC7466 were grown at 37°C in C + Y medium till OD_550 _reaching 0.1. Then 200 μl of the suspending bacteria was extracted into the wells of a 96-well plate for incubation at 37°C with the additions of 3 different dilutions of the 6 compounds. Subsequently, the plate was detected by spectrophotometer per hour for drawing the time- and concentration-dependent curve. All samples were assayed in triplicate, and each assay was repeated at least three times.

### *In vitro *cytotoxicity

Cytotoxicity of the antibacterial compounds on cultured Vero cell was measured by using the Cell Proliferation Kit I (MTT) (Sigma). Briefly, a series of dilution of the compounds were added into the medium, containing 1% of DMSO, to culture Vero cell. Cytotoxicity of the different concentration of chemicals was determined according to the kit protocol. Each assay was performed in quadruplicate and repeated three times. The results were converted to percentages of the control (cells only treated with 1% DMSO) and CC_50 _(concentrations that produce a 50% cytotoxicity effect on Vero cell) was calculated by using the SPSS 11.0 software.

### *In vivo *assays

Male and female BALB/c mice, aged 6–8 weeks (approx. 18–20 g), were used to evaluate the *in vivo *effects of the compounds. Briefly, these mice were randomly assigned to 8 groups (10-12 per group, half in each sex): 6 compound-treated groups, one negative control and one positive control. All the mice were administrated with 100 μl suspended *S. pneumoniae *strain ATCC 7466 (5 × 10^3 ^CFU/ml in phosphate buffered saline) by intraperitoneal injection route. Compounds (1–6) were diluted to the concentration of MIC respectively (1.27 mg/kg/d, 0.65 mg/kg/d, 1.13 mg/kg/d, 2.32 mg/kg/d, 1.27 mg/kg/d, 0.014 mg/kg/d, respectively) with normal sodium and 200 μl was administered by vena caudalis route after infection. Two control groups were administered with 200 μl normal sodium (negative control) and penicillin (0.42 mg/kg/d, positive control) respectively by the same injection route. Treatments were continued 3 times a day for 3 consecutive days, and these levels of chemicals caused few toxic influences on normal mice. The results are expressed as cumulative survival rates over the following 8-day observation.

## Authors' contributions

XZ and YY conceived of the study and participated in its design and coordination. NL, FW and WZ carried out the modeling of VicK protein and structure-based virtual screening. NL, SN, YL, KW and JC participated in the biological experiments of the *in vivo *assays and the *in vitro *assays. NL, FW and NY participated in analyzed the data and produced figures. NL, FW, WZ, XZ and YY drafted the manuscript. All the authors have read and approved the final manuscript.
